# Long noncoding RNA PPP1R14B-AS1 imitates microRNA-134-3p to facilitate breast cancer progression by upregulating LIM and SH3 protein 1

**DOI:** 10.32604/or.2022.03582

**Published:** 2022-08-31

**Authors:** LIMIN ZHOU, LIANBO ZHANG, XIN GUAN, YI DONG, TAO LIU

**Affiliations:** 1 Department of Second Breast Surgery, Jilin Cancer Hospital, Jilin, 130012, China; 2 Medical Insurance Guarantee Office, Jilin Cancer Hospital, Jilin, 130012, China; 3 Department of Breast Surgery, The First Hospital of Jilin University, Jilin, 130061, China

**Keywords:** Therapeutic target, ceRNA theory, Precision therapy, miRNA sponge

## Abstract

Long noncoding RNA PPP1R14B antisense RNA 1 (PPP1R14B-AS1) has emerged as a critical modulator of liver cancer and lung adenocarcinoma progression. However, the functional importance and biological relevance of PPP1R14B-AS1 in breast cancer remain unclear. Therefore, this study was designed to detect PPP1R14B-AS1 levels in breast cancer cells using qRT–PCR and elucidate the influence of PPP1R14B-AS1 on aggressive phenotypes. Furthermore, molecular events mediating the action of PPP1R14B-AS1 were characterized in detail. Functional experiments addressed the impacts of PPP1R14B-AS1 knockdown on breast cancer cells. In this study, PPP1R14B-AS1 was found to be overexpressed in breast cancer, exhibiting a close correlation with poor patient prognosis. Results also showed that breast cancer cell proliferation and motility were suppressed when PPP1R14B-AS1 was silenced. Mechanistically, PPP1R14B-AS1 acted as a competing endogenous RNA for microRNA-134-3p (miR-134-3p) in breast cancer cells. PPP1R14B-AS1 also increased LIM and SH3 protein 1 (LASP1) levels by imitating miR-134-3p in breast cancer cells. Rescue experiments further corroborated that the knockdown of miR-134-3p or an increase in LASP1 restored the aggressive malignant characteristics of breast cancer cells that were weakened by PPP1R14B-AS1 depletion. In summary, PPP1R14B-AS1 facilitated the oncogenicity of breast cancer cells by controlling the miR-134-3p/LASP1 axis. We believe that our findings may contribute to the development of precision therapy techniques in the field of breast cancer treatment.

## Introduction

Breast cancer ranks as the most frequently diagnosed human cancer and the leading cause of death among women worldwide [1]. According to the International Agency for Research on Cancer’s GLOBOCAN 2018, 2.1 million novel breast cancer cases were reported, and 0.63 million patients died of this cancer type globally [1]. At present, advances in anticancer therapies have notably improved the clinical outcomes of breast cancer [2]. However, patients with advanced-stage breast cancer still present unfavorable curative effects due to their malignant characteristics [3]. Moreover, multiple risk factors contribute to breast cancer tumorigenesis and progression [4]; nevertheless, detailed mechanisms are still not fully elucidated. Consequently, the development of promising diagnostic biomarkers and new therapeutic targets is urgently required for the early intervention and diagnosis of breast cancer.

Long noncoding RNAs (lncRNAs) are characterized as a group of RNA transcripts containing over 200 nucleotides [5]. Although lncRNAs possess no protein-coding ability, they are emerging as important players in gene silencing, transcriptional activation, chromosome modification, and intranuclear transport [6,7]. The contribution of lncRNAs in breast cancer has, therefore, received increasing attention [8–10]. Furthermore, many studies have provided evidence supporting lncRNA dysregulation in breast cancer and their significant roles [11–13]. For instance, LRP11-AS1 [14], LINC000466 [15] and LINC00649 [16] are highly expressed in breast cancer cells and confirmed to be oncogenic lncRNAs. In contrast, the downregulation of MBNL1-AS1 [17] and LINC00921 [18] in breast cancer cells has been reported to exert antitumor activities.

MicroRNAs (miRNAs) are short single-stranded noncoding RNA transcripts typically composed of 17–22 nucleotides [19]. They have emerged as critical posttranscriptional regulators that act by directly binding to the 3′-UTRs of their targets and, consequently, triggering mRNA degradation or translation restriction [20]. Therefore, the crosstalk between lncRNAs and miRNAs has been studied comprehensively. Additionally, a competing endogenous RNA (ceRNA) theory has been proposed, which revealed that lncRNAs can adsorb miRNAs and thereby lower the modulatory actions of miRNAs on downstream targets [21–24]. Consequently, illustrating the detailed functions of lncRNAs and miRNAs is proposed to be helpful for clinical diagnosis and provision of alternative treatment options for breast cancer.

Alternatively, PPP1R14B-AS1 has emerged as a critical controller of liver cancer and lung adenocarcinoma progression [25]. However, its role in breast cancer has not been defined yet. Thus, this study was designed to detect PPP1R14B-AS1 levels in breast cancer cells and elucidate its influence on them. Furthermore, molecular events mediating the action of PPP1R14B-AS1 were characterized in detail.

## Materials and Methods

### Clinical specimens

The Ethics Committee of The First Hospital of Jilin University approved our research. First, breast cancer tissues and adjacent healthy tissues were obtained from 41 patients in our hospital with breast cancer. The inclusion criteria were as follows: (i) patients diagnosed with breast cancer; (ii) those previously treated with radiotherapy, chemotherapy, or other types of anticancer treatments; and (iii) those who agreed to participate in the research. The exclusion criteria were as follows: (i) patients receiving radiotherapy or chemotherapy, (ii) those with other types of cancer, and (iii) those who refused to participate in the research. RNALater™ RNA Stabilization Reagent (Beyotime; Shanhgai, China) was used, and all clinical samples were stored in liquid nitrogen until further use. Additionally, all participants signed informed consent forms before they were enrolled in the study.

### Cell culture

A normal human immortalized breast epithelial cell line MCF-10A (ATCC, Rockville, MD, USA) was cultured in MEGM^TM^ Mammary Epithelial Cell Growth Medium BulletKit^TM^ (Lonza/Clonetics Corporation, Walkersville, MD, USA) that was supplemented with 100 ng/ml cholera toxin. The Type Culture Collection of the Chinese Academy of Sciences (Shanghai, China) is the provider of all breast cancer cell lines. Cell lines BT-549 was maintained in Roswell Park Memorial Institute Medium 1640 containing 10% fetal bovine serum (FBS), while 10% FBS-supplemented L-15 medium (all from Gibco, Grand Island, NY, USA) was used for the culture of cell lines MDA-MB-468 and MDA-MB-231. Minimum Essential Medium (Gibco) was added with 1% Glutamax, 1% non-essential amino acids, 1% sodium pyruvate 100 mM solution for culturing MCF-7 cells. All cells were cultured at 37°C in a humidified incubator under 5% CO_2_.

### Cell transfection

For gene silencing studies, small interfering RNA (siRNA) against PPP1R14B-AS1 (si-PPP1R14B-AS1) and negative control siRNA (si-NC) were bought from GenePharma (Shanghai, China). Then, for high mobility group box 3 (LASP1) overexpression plasmid generation, LASP1 sequences were inserted into the pcDNA3.1 + vector, yielding the pcDNA3.1-LASP1 plasmid. MiR-134-3p mimic, NC mimic, miR-134-3p inhibitor (anti-miR-134-3p), and NC inhibitor (anti-NC) were all designed and synthesized by RiboBio (Guangzhou, China). Furthermore, lipofectamine 2000 reagent (Invitrogen; Thermo Fisher Scientific, Inc., Waltham, MA, USA) was used for transfection experiments.

### Quantitative real-time polymerase chain reaction (qRT–PCR)

Total RNA was isolated using TRIzol (Invitrogen). The reverse transcription step was performed using miRcute miRNA First-Strand cDNA Synthesis Kit (Tiangen, Beijing, China) to determine miRNA expression. Next, the miRcute miRNA qPCR Detection Kit (Tiangen) was employed for the quantification of miRNA.

For detecting PPP1R14B-AS1 and LASP1 levels, the PrimeScript™ RT reagent kit and TB Green® Premix Ex Taq™ II (both from Takara, Dalian, China) were employed for reverse transcription and PCR amplification. Furthermore, U6 and GAPDH were used to normalize miRNA and mRNA (lncRNA) expression, respectively. Finally, all data were analyzed using the 2^−ΔΔCt^ method.

### Subcellular localization analysis

Nuclear and cytosolic RNAs were isolated from breast cancer cells using a cytoplasmic and nuclear RNA Purification Kit (Norgen Biotek Corp., Thorold, ON, Canada). qRT–PCR was then used to determine the relative abundance of PPP1R14B-AS1 in nuclear and cytosolic RNAs.

### Cell counting kit-8 (CCK-8) and colony formation assays

Transfected cells were inoculated into 96-well plates at a density of 2 × 10^3^ cells per well. Each group contained five replicate wells. After the cells were cultivated for different time periods, 10 µl of the CCK-8 solution (Dojindo Molecular Technologies, Inc., Kumamoto, Japan) was added, and the cells were incubated at 37°C for an additional 2 h. Subsequently, a microplate reader was used to determine the absorbance at 450-nm wavelength. Next, the average absorbance value for each group of replicate wells was analyzed.

For colony formation assay, 2 ml culture medium containing 500 cells was added into 6-well plates. Subsequent to 2 weeks cultivation at 37°C with 5% CO_2_, the newly formed colonies were fixed with 4% paraformaldehyde, followed by staining with 0.1% crystal violet. After extensive washing, the number of colonies was counted applying an inverted light microscope (Olympus, Tokyo, Japan).

### Transwell migration and invasion experiments

Transwell chambers (8-μm pore size) precoated with Matrigel® (BD Biosciences, Franklin Lakes, NJ, USA) were used to detect cell invasive capacity. Briefly, a cell suspension was produced using a serum-free culture medium. Then, while 600 µl of 10% FBS-containing culture medium was seeded into the lower compartments, the upper chambers were inoculated with 200 μl of cell suspension containing 5 × 10^4^ cells. Subsequently, the transferred cells were cultivated at 37°C for the whole day. After removing the noninvading cells, 4% paraformaldehyde and 0.1% crystal violet were used to immobilize and stain the invading cells. Next, after extensive washing, the invading cells were viewed and counted under an inverted light microscope. The migration experiment was performed in accordance with the abovementioned experimental procedures, except that Matrigel® was not used.

### Subcutaneous tumor formation in nude mice

*In vivo* experiments were conducted after obtaining approval from the Animal Research Ethics Committee of The First Hospital of Jilin University. GenePharma designed and synthesized short-hairpin RNA (shRNA) targeting PPP1R14B-AS1 (sh-PPP1R14B-AS1) and the nontarget control shRNA (sh-NC). Afterward, the manufactured shRNAs were inserted into the pLKO.1 vector (Addgene, Inc., Watertown, MA, USA), and the generated vectors were then transfected into 293T cells alongside a lentiviral packaging plasmid psPAX2 and a envelope expression plasmid pMD2.G. Two days later, we harvested the lentiviruses by ultracentrifugation (1,000 × g), which were subsequently used to infect MCF-7 cells. Additionally, puromycin was employed for selecting MCF-7 cells with stable expression of sh-PPP1R14B-AS1 or sh-NC.

BALB/C female nude mice aged 4–6 weeks (SLAC Laboratory Animal, Co., Ltd., Shanghai, China) were employed for developing xenograft tumors. First, mice were injected subcutaneously with 1 × 10^6^ MCF-7 cells with stable sh-PPP1R14B-AS1 or sh-NC expression. Each group had three nude mice. Then, tumor width (W) and length (L) were recorded every 5 days, and the acquired data was used for calculating tumor volume using the following formula: V = 0.5 × L × W^2^. On day 30, all mice were euthanized using the cervical dislocation method. Next, the xenograft tumors were resected from nude mice, weighed, and stored until further analysis.

### Bioinformatics prediction

PPP1R14B-AS1 expression in breast invasive carcinoma from TCGA samples was analzyed utilizing UALCAN (http://ualcan.path.uab.edu). Potential target miRNAs of PPP1R14B-AS1 were predicted using miRDB [26] (http://mirdb.org/). Alternatively, TargetScan [27] (http://www.targetscan.org/vert_60/) and miRDB were used to identify the direct target of miR-134-3p.

## Luciferase Reporter Assay

GenePharma amplified PPP1R14B-AS1 and LASP1 fragments carrying the wild-type (wt) miR-134-3p binding sequences and inserted them into a psiCHECK™-2 vector (Promega, Madison, WI, USA), generating the PPP1R14B-AS1-wt and LASP1-wt reporter plasmids. Subsequently, the PPP1R14B-AS1-mutant (PPP1R14B-AS1-mut) and LASP1-mut reporter plasmids were yielded in the same way. Then, reporter plasmids and miR-134-3p mimic or NC mimic were introduced into breast cancer cells, followed by a 48-h incubation at 37°C. Finally, the activity of the reporter was assayed using a Dual-Luciferase Reporter Assay System (Promega).

### RNA immunoprecipitation (RIP)

Breast cancer cells were collected and lysed in an equal volume of RIP buffer. The assay was performed using a Millipore Magna RIP™ RNA-Binding Protein Immunoprecipitation Kit (Merck-Millipore, Bedford, MA, USA). First, a 100 µl aliquot of cell lysate was added to 900 μL of RIP immunoprecipitation buffer, followed by further cultivation overnight at 4°C with magnetic beads conjugated with human anti-Ago2 or control anti-IgG antibodies (Merck-Millipore). After digestion with proteinase K, the immunoprecipitated RNA was further analyzed using qRT–PCR.

### Western blotting

RIPA reagent (Sigma, St. Louis, MO, USA) was employed for total protein isolation. First, after quantification using bicinchoninic acid protein assay kit (Beyotime), proteins were electrophoresed using a 10% SDS–PAGE electrophoresis protocol. Then, separated proteins were transferred to polyvinylidene difluoride membranes before blocking them with 5% nonfat dried milk. Thereafter, primary antibodies against LASP1 (ab156872; Abcam, Cambridge, UK) and GAPDH (ab204481; Abcam) were added, and the solution was incubated overnight at 4°C. Next, tris-buffered saline with Tween buffer was used to clean the membranes and then HRP-conjugated secondary antibody (ab205718; Abcam) was applied and incubated at room temperature for 2 h. Finally, protein signals were detected using an ECL Western blotting Substrate Kit (Abcam).

### Statistical analysis

Functional experiments were repeated three times independently (obtained data are shown as the mean ± standard deviation). A normality test was performed utilizing Shapiro–Wilk normality test. The overall survival of the patients with breast cancer was estimated using Kaplan–Meier analysis and compared using the log-rank test. All statistical evaluations were also conducted using the Student’s *t* test or analysis of variance with Tukey’s post hoc test. The SPSS statistical software package (standard version 18.0, SPSS Inc., Chicago, IL, USA) was employed for all statistical analyses, and *p* < 0.05 was considered as statistically significant.

## Results

### Loss of PPP1R14B-AS1 abates the malignant phenotype of bresat cancer cells

**Figure 1:**
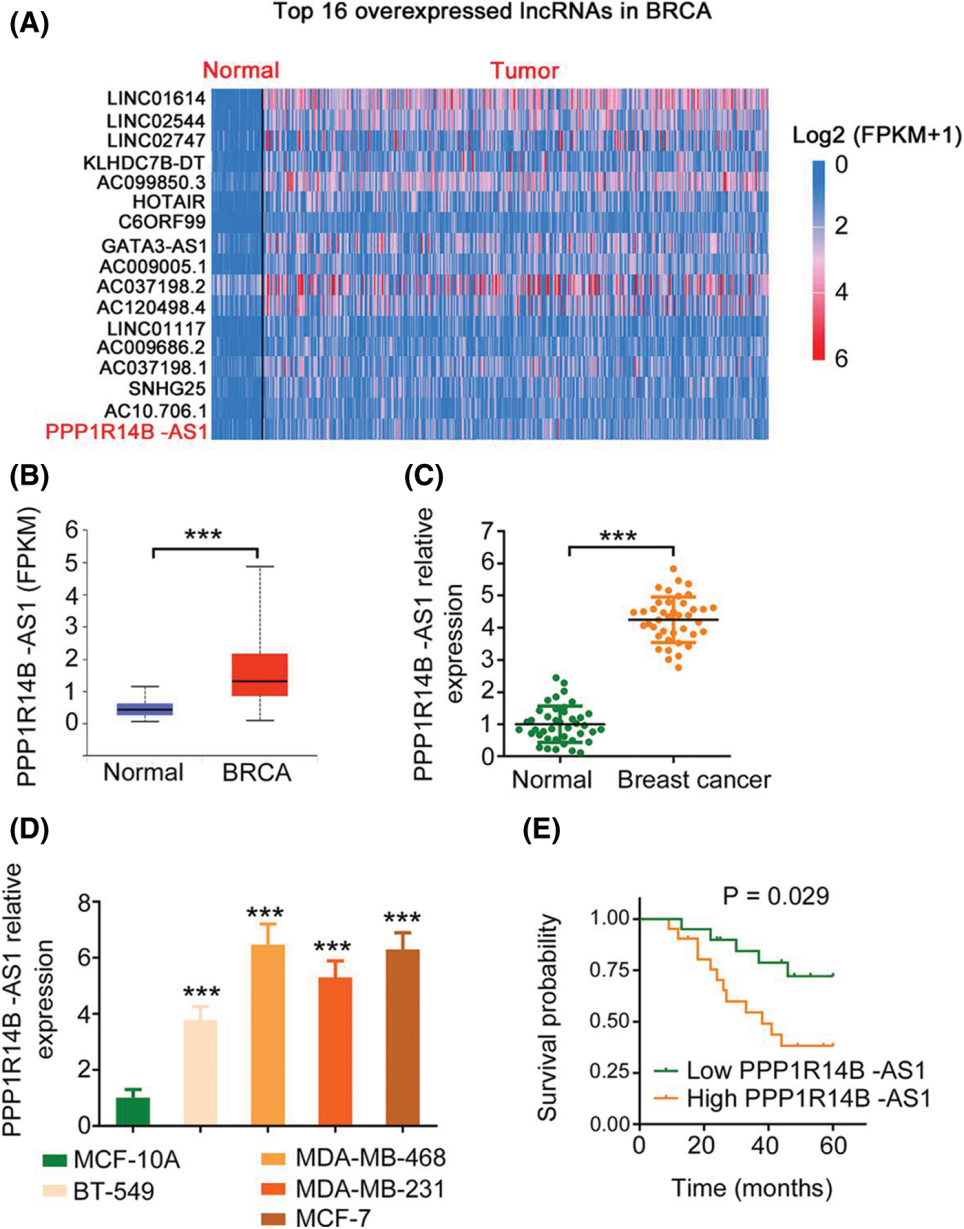
PPP1R14B-AS1 is overexpressed in breast cancer. (A) PPP1R14B-AS1 ranks 16^th^ among overexpressed lncRNAs in BRCA samples from TCGA dataset. (B) The TCGA database was analyzed to examine PPP1R14B-AS1 expression in BRCA tissue samples. (C) qRT–PCR results showing expression levels of PPP1R14B-AS1 in breast cancer tissue samples from our own cohort. (D) qRT–PCR results showing PPP1R14B-AS1 expression in different breast cancer cell lines. (E) The correlation between PPP1R14B-AS1 levels and survival rates of patients with breast cancer. ***p < 0.001 (n = 3).

First, utilizing TCGA database, we found that PPP1R14B-AS1 ranks 16^th^ among overexpressed lncRNAs in breast invasive carcinoma (BRCA; Fig. 1A). Next, 41 breast cancer tissues and adjacent healthy tissues were collected and used to verify the expression status of PPP1R14B-AS1. Consistent with the TCGA results (Fig. 1B), PPP1R14B-AS1 was highly expressed in breast cancer tissues compared with adjacent healthy normal tissues (Fig. 1C). Similarly, PPP1R14B-AS1 was overexpressed in breast cancer cell lines compared with the normal human immortalized breast epithelial cell line MCF-10A (Fig. 1D). All patients were divided into high or low PPP1R14B-AS1 groups based in the median value of PPP1R14B-AS1 in tumor tissues. Results also showed that patients with breast cancer having high PPP1R14B-AS1 levels had shorter lifespans than those having low PPP1R14B-AS1 levels (Fig. 1E).

**Figure 2:**
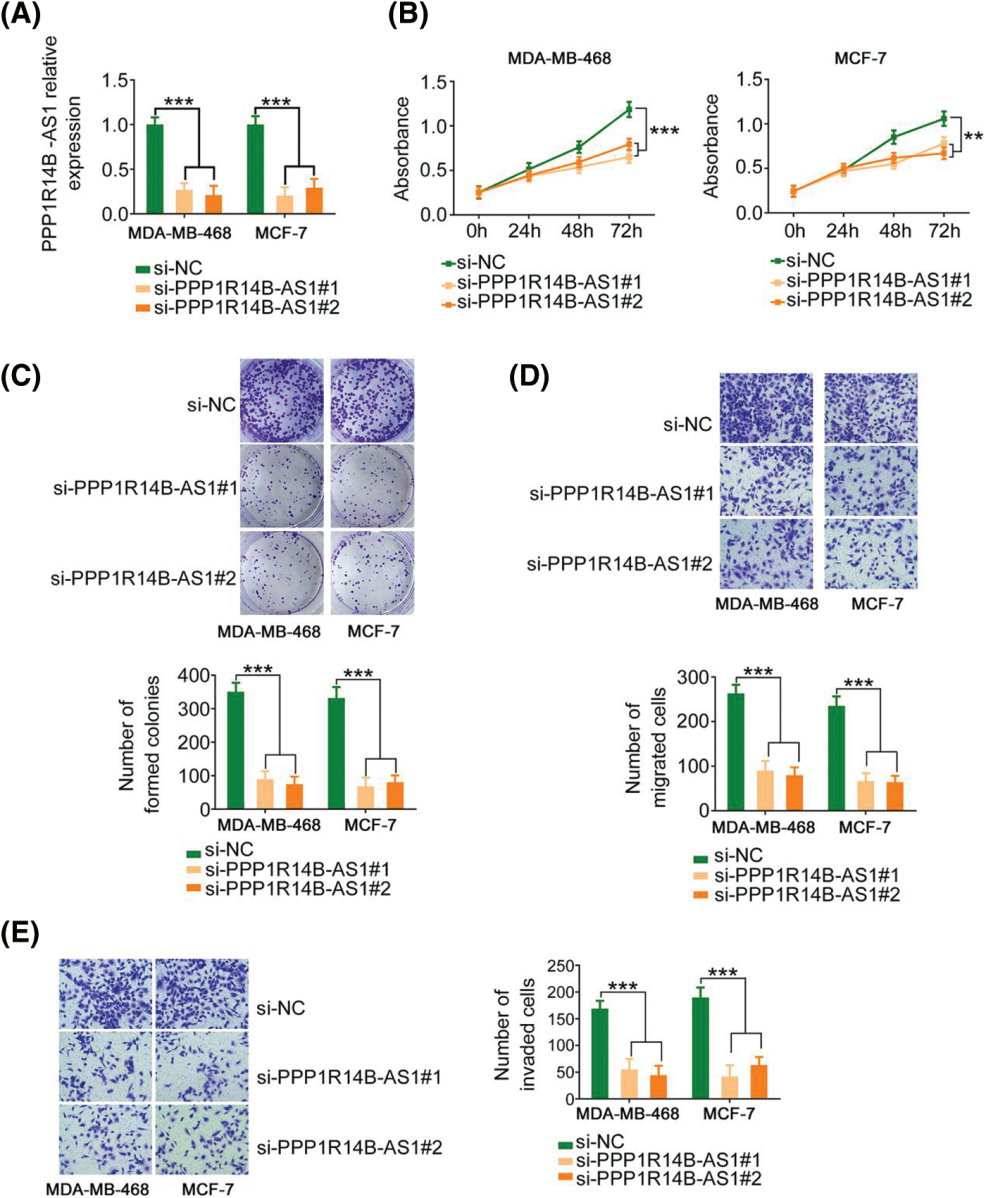
PPP1R14B-AS1 silencing inhibits the malignant progression of breast cancer cells *in vitro*. (A) qRT–PCR results confirming the transfection efficiency of si-PPP1R14B-AS1. (B, C) CCK-8 and colony formation assays results revealing the change in the proliferation of breast cancer cells after si-PPP1R14B-AS1 transfection. (D, E) Effects of PPP1R14B-AS1 silencing on the migratory and invasive abilities of breast cancer cells. 100× magnification. **p < 0.01 and ***p < 0.001 (n = 3).

Therefore, to evaluate the effect of PPP1R14B-AS1 on breast cancer cells, MDA-MB-468 and MCF-7 cell lines were chosen for functional assays and transfected with si-PPP1R14B-AS1 to lower PPP1R14B-AS1 expression. Then, qRT–PCR was used to verify the successful knockdown of PPP1R14B-AS1 by si-PPP1R14B-AS1 (Fig. 2A). As observed, after PPP1R14B-AS1 depletion, breast cancer cells grew at a slower rate (Figs. 2B and 2C). Furthermore, fewer PPP1R14B-AS1-silenced breast cancer cells passed through the pores in the Transwell chambers precoated with or without Matrigel, implying that PPP1R14B-AS1 knockdown hindered migratory and invasive (Figs. 2D and 2E) capacities. Thus, PPP1R14B-AS1 was observed to be a pro-oncogenic lncRNA found in breast cancer cells and involved in tumor progression.

### PPP1R14B-AS1 binds directly to and acts as a sponge of miR-134-3p in breast cancer cells

**Figure 3:**
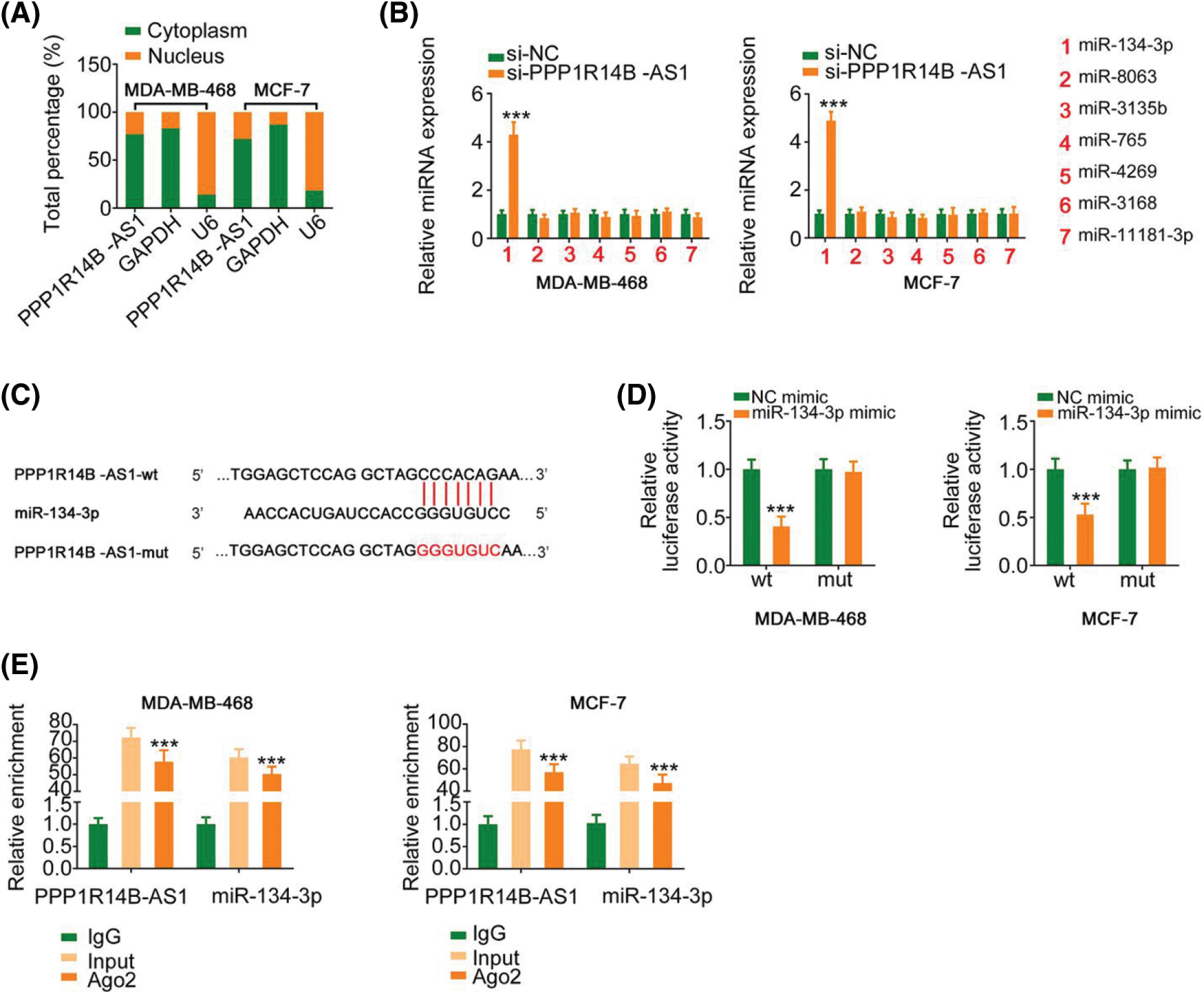
PPP1R14B-AS1 acts as an miR-134-3p sponge. (A) Relative distribution of PPP1R14B-AS1 in nuclear and cytosolic fractions of breast cancer cells was detected via subcellular localization analysis. (B) qRT–PCR results showing the levels of the seven candidate molecules in breast cancer cells after PPP1R14B-AS1 depletion. (C) The predicted miR-134-3p binding site within PPP1R14B-AS1. Mutated binding sequences are underlined. (D) Luciferase reporter assay results showing the luciferase activity of breast cancer cells upon cotransfection with miR-134-3p mimic or NC mimic and PPP1R14B-AS1-wt or PPP1R14B-AS1-mut. (E) The relationship between PPP1R14B-AS1 and miR-134-3p in breast cancer cells as assessed by the RIP assay. ***p < 0.001 (n = 3).

To decipher the molecular events by which PPP1R14B-AS1 exerts its cancer-promoting activity, subcellular localization analysis was performed to test the location of PPP1R14B-AS1 in breast cancer cells. Results proved that PPP1R14B-AS1 was abundant in the nucleus and cytoplasm, with the latter harboring a larger portion (Fig. 3A). This observation proposes that PPP1R14B-AS1 can affect breast cancer progression based on ceRNA levels. miRDB is a publicly available bioinformatic algorithm and functional annotations. By searching miRDB, 15 miRNAs were predicted to possess potential binding sites for PPP1R14B-AS1 (Table 1). Among these candidates, miR-134-3p [28], miR-8063 [29], miR-3135b [30], miR-765 [31], miR-4269 [32], miR-3168 [33] and miR-11181-3p [34] were reported to contribute to carcinogenesis and cancer progression; thus, they were chosen for further verification. Afterward, qRT–PCR was conducted to quantify these miRNAs in si-PPP1R14B-AS1-transfected breast cancer cells. We observed that although miR-134-3p was significantly increased in PPP1R14B-AS1-silenced breast cancer cells, levels of other miRNAs were unaffected (Fig. 3B).

**Table 1:** The 15 predicted miRNAs targeting PPP1R14B-AS1

hsa-miR-8063	hsa-miR-6715b-5p	hsa-miR-4269
hsa-miR-4774-3p	hsa-miR-3135b	hsa-miR-3168
hsa-miR-12119	hsa-miR-765	hsa-miR-4303
hsa-miR-6811-5p	hsa-miR-4318	hsa-miR-11181-3p
hsa-miR-4519	hsa-miR-134-3p	hsa-miR-6511b-5p

The predicted binding site between miR-134-3p and PPP1R14B-AS1 is presented in Fig. 3C. Moreover, the binding interaction was subsequently verified. As observed, although miR-134-3p overexpression downregulated the luciferase activity in PPP1R14B-AS1-wt, it had no such effects in PPP1R14B-AS1-mut (Fig. 3D). Interestingly, PPP1R14B-AS1 and miR-134-3p were abundant in products immunoprecipitated by anti-Ago2 antibody (Fig. 3E), suggesting a direct interaction between the two RNAs. These results indicated that PPP1R14B-AS1 directly targets miR-134-3p and acts as its sponge in breast cancer cells.

### miR-134-3p directly targets LASP1 in breast cancer cells

**Figure 4:**
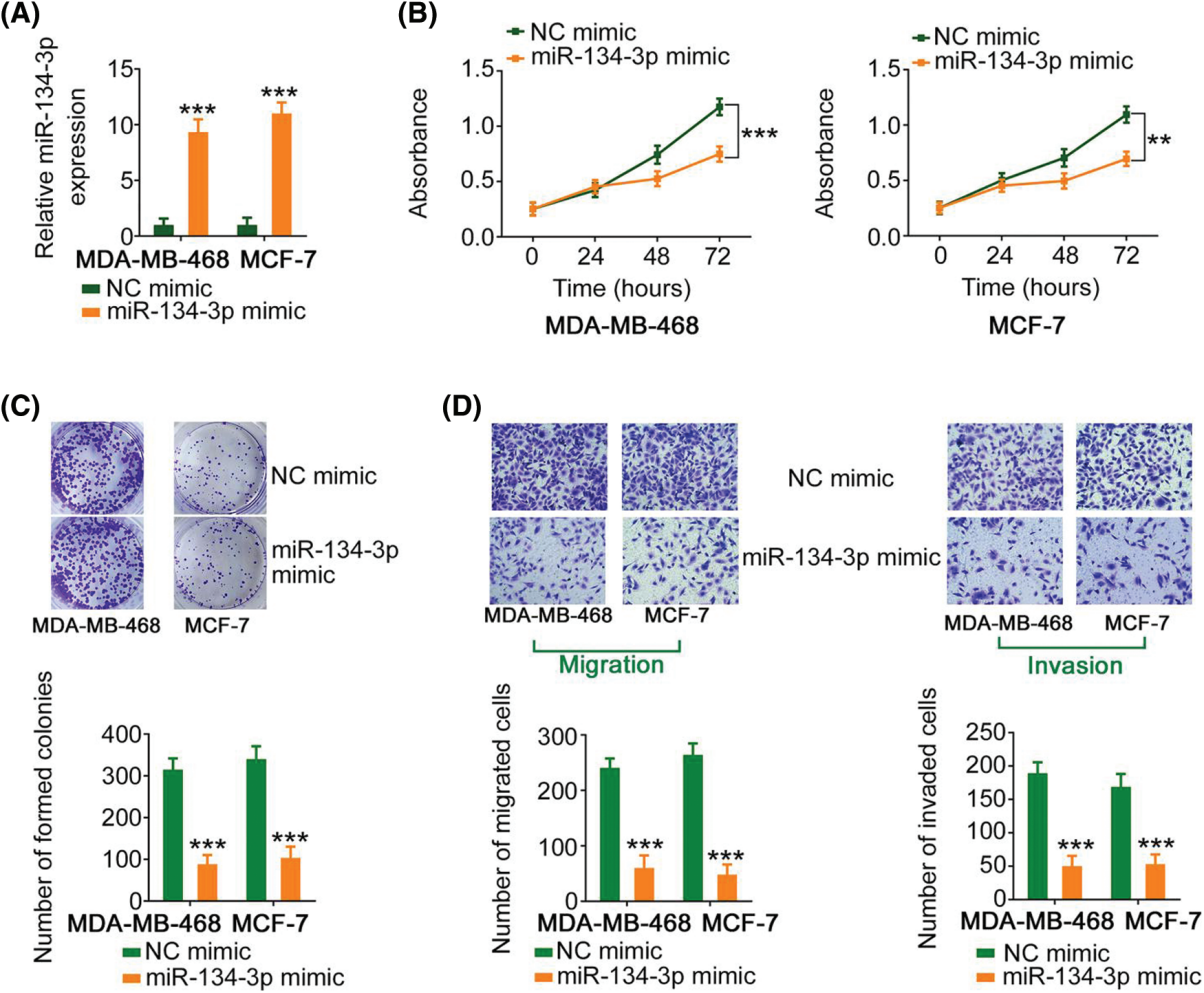
miR-134-3p suppresses the malignancy of breast cancer cells. (A) The efficiency of miR-134-3p mimic transfection into breast cancer cells was investigated. (B, C) The proliferation of miR-134-3p-overexpressing breast cancer cells was evaluated. (D) The migration and invasion of miR-134-3p-overexpressing breast cancer cells. 100× magnification. **p < 0.01 and ***p < 0.001 (n = 3).

Since miR-134-3p was downregulated in breast cancer cells, its detailed effects on breast cancer cell behavior were also examined. The upregulation of miR-134-3p by miR-134-3p mimic (Fig. 4A), attenuated the proliferative capacity of breast cancer cells (Figs. 4B and 4C). Furthermore, ectopic miR-134-3p expression strikingly decreased the motility (Fig. 4D) of breast cancer cells.

**Figure 5:**
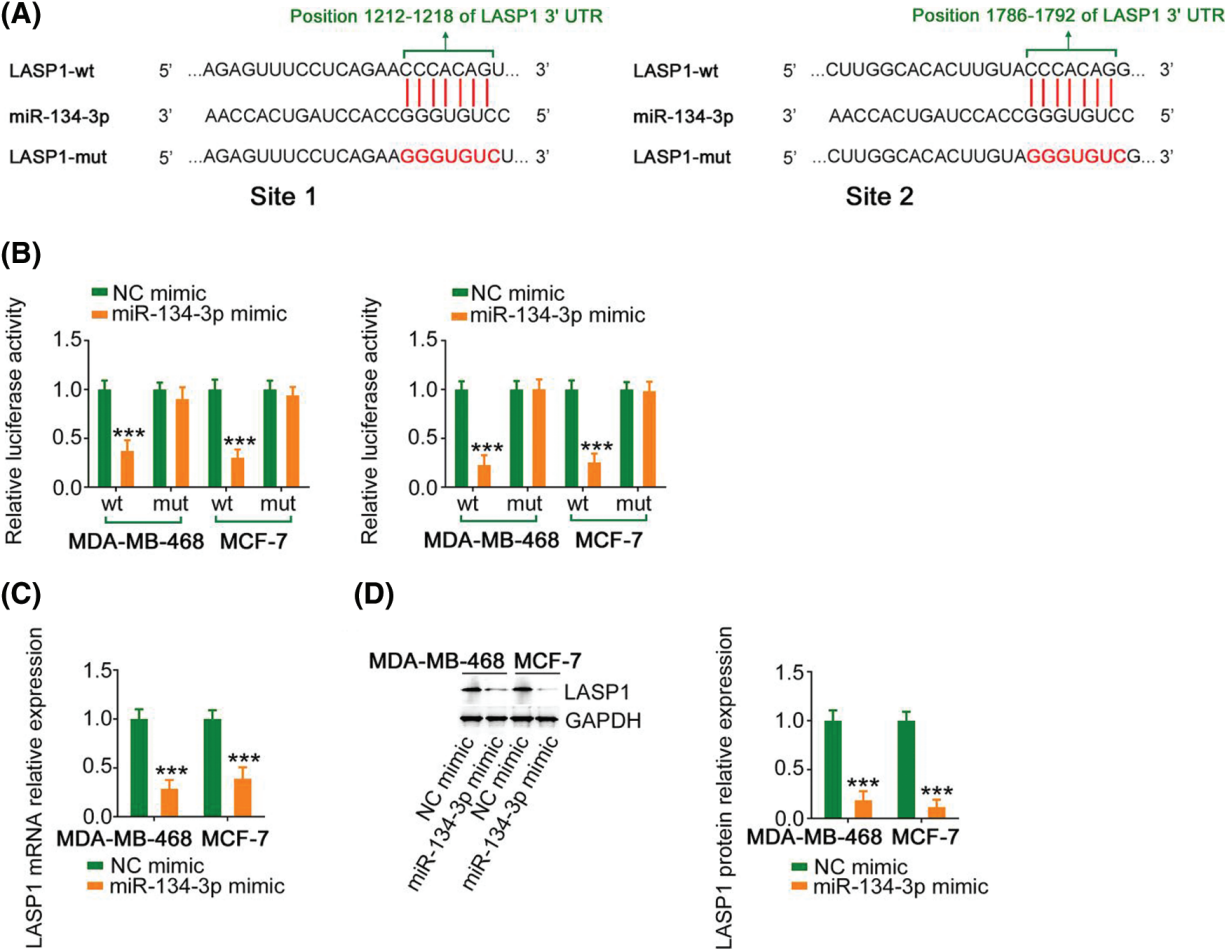
Verification of LASP1 as a direct target of miR-134-3p. (A) The predicted miR-134-3p binding sequences within the 3′-UTR of LASP1. (B) A luciferase reporter assay showing the binding interaction between miR-134-3p and LASP1. (C, D) LASP1 expression in breast cancer cells was measured when miR-134-3p was overexpressed. ***p < 0.001 (n = 3).

Using bioinformatics analysis, LASP1 was predicted as a candidate molecule that regulates miR-134-3p (Fig. 5A) and was selected for research due to its considerable regulatory actions toward breast cancer genesis and progression. In breast cancer cells, cotransfection with miR-134-3p mimic lowered the activity of LASP1-wt; however, such influences were counteracted after the binding sequences were mutated (Fig. 5B). Furthermore, miR-134-3p overexpression evidently lowered LASP1 expression in breast cancer cells (Figs. 5C and 5D). Altogether, the above results indicate that antioncogenic miR-134-3p directly targets LASP1 in breast cancer cells.

### The PPP1R14B-AS1/miR-134-3p axis regulates LASP1 expression in breast cancer cells

**Figure 6:**
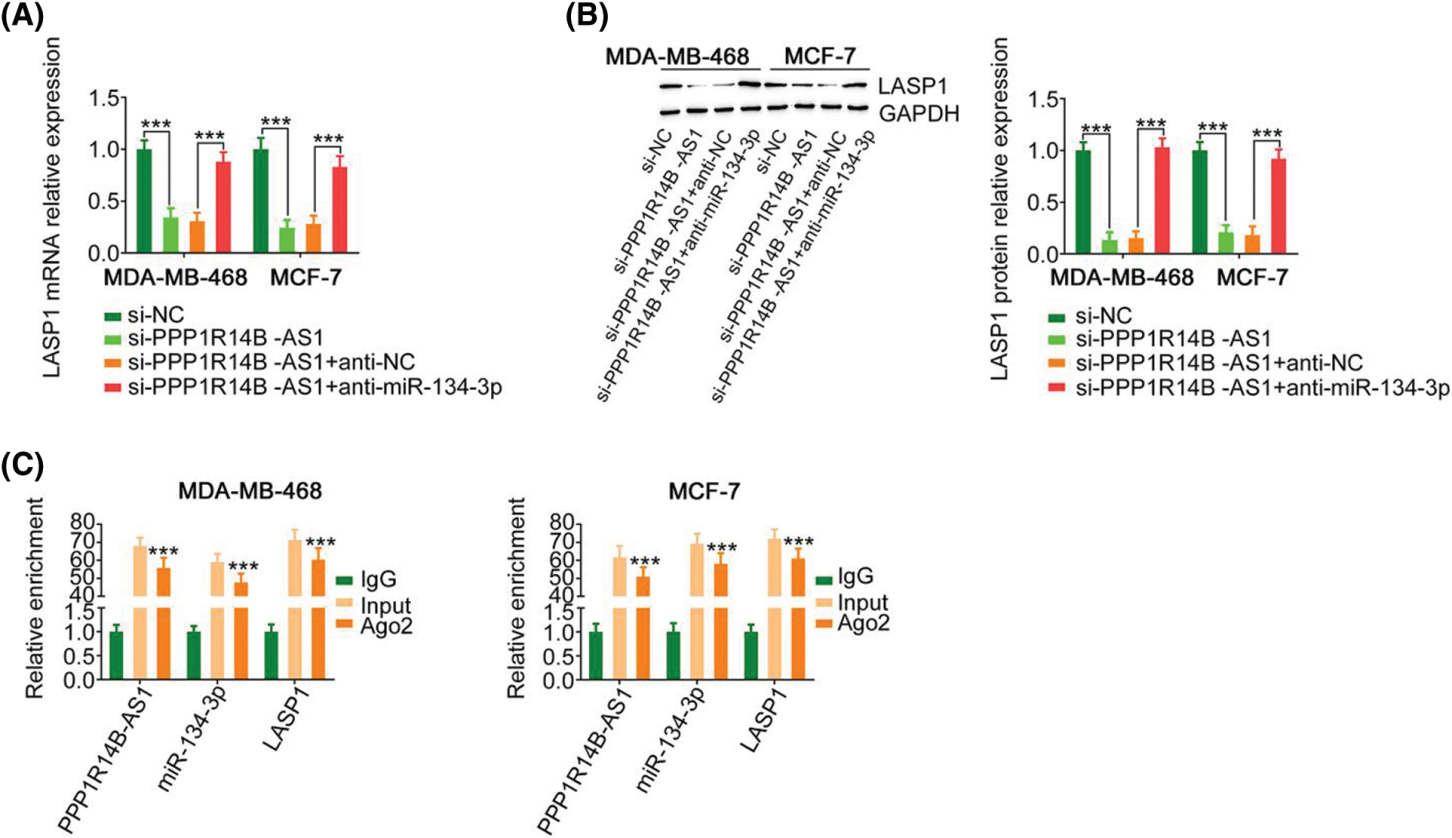
PPP1R14B-AS1 indirectly regulated LASP1 expression by functioning as a ceRNA of miR-134-3p. (A, B) Breast cancer cells were transfected with si-PPP1R14B-AS1 in parallel with anti-miR-134-3p, followed by the measurement of LASP1 levels. (C) The relationship between PPP1R14B-AS1, miR-134-3p and LASP1 in breast cancer cells as assessed by the RIP assay. ***p < 0.001 (n = 3).

Since we identified a relationship between miR-134-3p; its upstream regulator, PPP1R14B-AS1; and its downstream target, LASP1, the following experiments were performed to uncover the regulatory associations between PPP1R14B-AS1, miR-134-3p, and LASP1 in breast cancer cells. The absence of PPP1R14B-AS1 led to the downregulation of LASP1 levels in breast cancer cells. However, such regulatory activity was counteracted by the cotransduction of anti-miR-134-3p (Figs. 6A and 6B). Furthermore, PPP1R14B-AS1, miR-134-3p and LASP1 were abundant in products immunoprecipitated by anti-Ago2 antibody (Fig. 6C) in breast cancer cells. Overall, results showed that PPP1R14B-AS1 acted as a ceRNA for miR-134-3p and consequently increased LASP1 levels in breast cancer cells.

### The miR-134-3p/LASP1 axis is essential for the actions of PPP1R14B-AS1 in breast cancer cells

**Figure 7:**
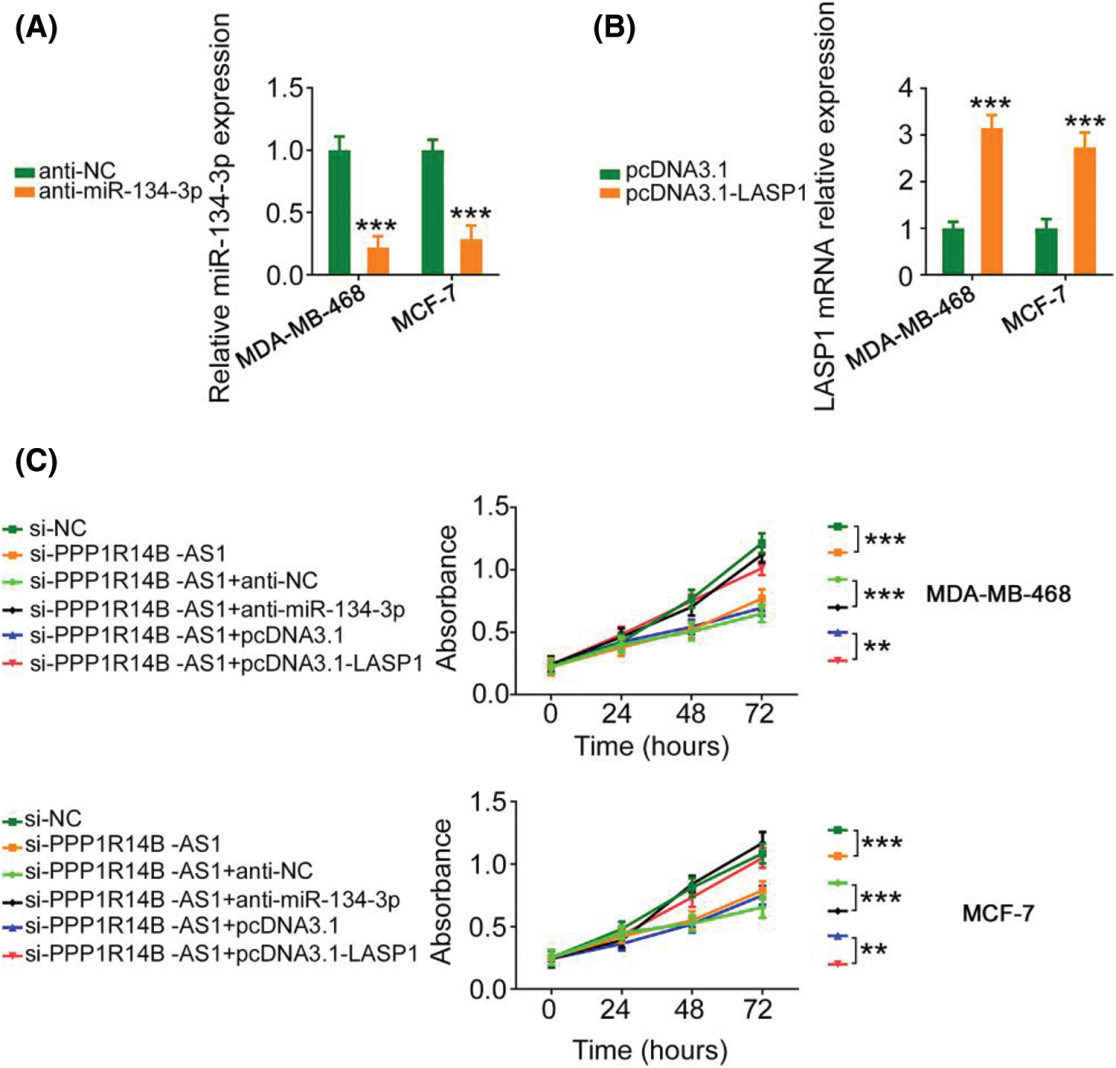
Anti-miR-134-3p or reintroduction of LASP1 counteracts the influence of si-PPP1R14B-AS1 on breast cancer cell proliferation. (A) miR-134-3p levels measured in breast cancer cells upon anti-miR-134-3p transfection. (B) LASP1 levels measured in breast cancer cells upon pcDNA3.1-LASP1 transfection. (C) Breast cancer cells were further treated with si-NC, si-PPP1R14B-AS1, si-PPP1R14B-AS1 + anti-NC, si-PPP1R14B-AS1 + anti-miR-134-3p, si-PPP1R14B-AS1 + pcDNA3.1, or si-PPP1R14B-AS1 + pcDNA3.1-LASP1. Cell proliferation change was determined after transfection. **p < 0.01 and ***p < 0.001 (n = 3).

**Figure 8:**
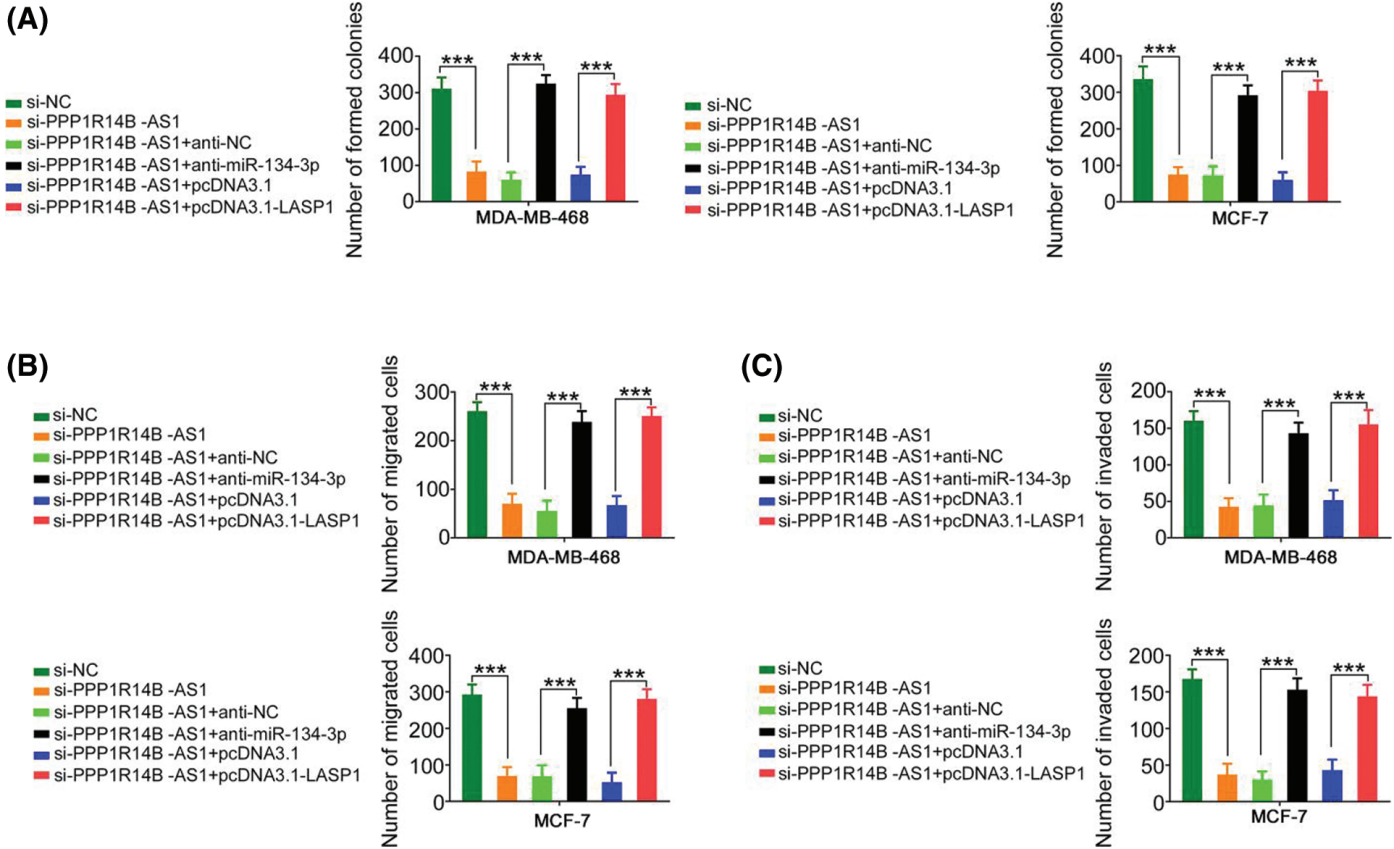
The regulatory effect of si-PPP1R14B-AS1 on the colony formation and motility of breast cancer cells was reversed by lowering miR-134-3p or overexpressing LASP1 levels. (A) PPP1R14B-AS1-depleted breast cancer cells were further cotransfected with anti-miR-134-3p or pcDNA3.1-LASP1. After transfection, colony formation assay was implemented to assess colony formative ability. (B, C) Breast cancer cells were transfected with si-NC, si-PPP1R14B-AS1, si-PPP1R14B-AS1 + anti-NC, si-PPP1R14B-AS1 + anti-miR-134-3p, si-PPP1R14B-AS1 + pcDNA3.1, or si-PPP1R14B-AS1 + pcDNA3.1-LASP1. Cell migration and invasion was detected utilizing Transwell migration and invasion experiments. ***p < 0.001 (n = 3).

The contribution of the miR-134-3p/LASP1 axis toward the oncogenic roles of PPP1R14B-AS1 in breast cancer cells was evaluated using rescue experiments. Anti-miR-134-3p decreased miR-134-3p levels (Fig. 7A), whereas the LASP1 overexpression plasmid, pcDNA3.1-LASP1, increased LASP1 levels in breast cancer cells (Fig. 7B). Furthermore, while the knockdown of PPP1R14B-AS1 repressed breast cancer cell proliferation (Fig. 7C) and colony formation (Fig. 8A), cotransfection with anti-miR-134-3p or pcDNA3.1-LASP1 offset the repressing effects. Similarly, treatment with anti-miR-134-3p or pcDNA3.1-LASP1 reversed the regulatory effect of si-PPP1R14B-AS1 on breast cancer cell migration (Fig. 8B) and invasion (Fig. 8C). The abovementioned results together confirmed that PPP1R14B-AS1 facilitated breast cancer cell malignancy by controlling the miR-134-3p/LASP1 axis.

### Suppression of PPP1R14B-AS1 impairs tumorigenesis of breast cancer cells in vivo

**Figure 9:**
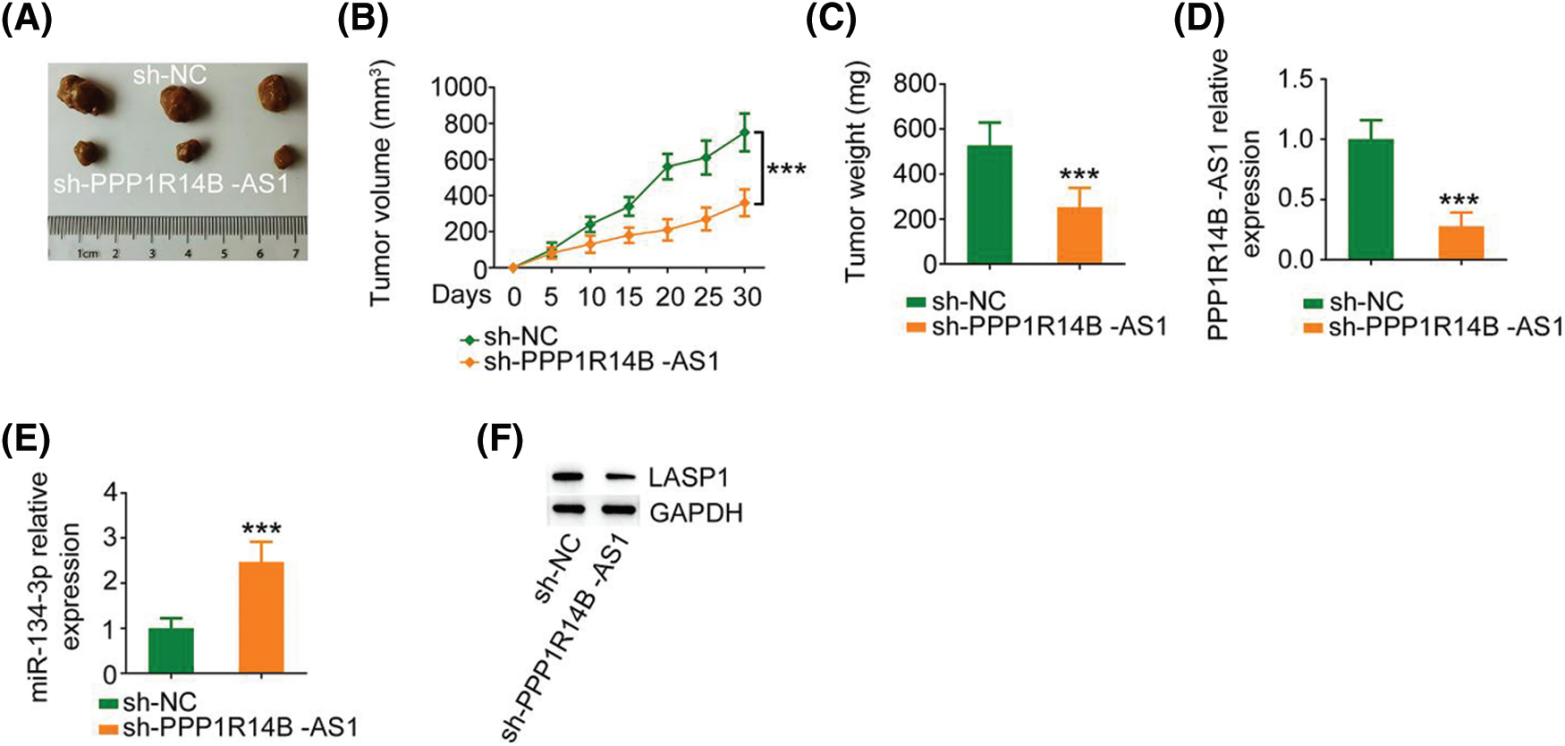
PPP1R14B-AS1 knockdown hinders tumor growth *in vivo*. (A) Images of xenograft tumors. (B) Tumor volume was recorded every 5 days to examine the growth of the tumors. (C) The weight of xenograft tumors. (D, E) PPP1R14B-AS1 and miR-134-3p levels in xenograft tumors. (F) The protein level of LASP1 in xenograft tumors. ***p < 0.001 (n = 3).

Finally, a subcutaneous tumor was generated in nude mice to investigate the carcinogenic roles of PPP1R14B-AS1 in breast cancer cells. Notably, the growth of xenograft tumors formed by MCF-7 cells stably expressing sh-PPP1R14B-AS1 was hindered compared to the growth of those formed by MCF-7 cells expressing sh-NC (Figs. 9A and 9B). Moreover, the weight of xenograft tumors resected on day 30 from the sh-PPP1R14B-AS1 group was lower than the weight of those resected from the sh-NC group (Fig. 9C). Subsequently, we performed qRT–PCR to quantify PPP1R14B-AS1 and miR-134-3p levels in xenograft tumors. A reduction in PPP1R14B-AS1 levels was noted in xenograft tumors isolated from mice injected with stable PPP1R14B-AS1-depleted MCF-7 cells (Fig. 9D), whereas the level of miR-134-3p formed by MCF-7 cells with ablated PPP1R14B-AS1 was enhanced (Fig. 9E). We also observed a notable decrease of LASP1 levels in tumors isolated from the sh-PPP1R14B-AS1 group (Fig. 9F). In short, the depletion of PPP1R14B-AS1 decreased LASP1 levels via the sequestration of miR-134-3p, consequently impairing tumor growth *in vivo*.

## Discussion

It has already been established that lncRNAs are important regulators and control complex cellular behavior in breast cancer [35–37], which has received much attention from several researchers. Based on the importance of lncRNAs in breast cancer, identifying novel breast cancer-related lncRNAs and elucidating their functions in tumorigenesis are of great value. However, the functional significance and biological relevance of several lncRNAs in breast cancer are still not clearly understood. Thus, this study evaluated the roles of PPP1R14B-AS1 in breast cancer and offered novel insights on anticancer therapies.

High PPP1R14B-AS1 expression has been reported in patients with liver cancer and lung adenocarcinoma [25]. PPP1R14B-AS1 has been shown to suppress liver cancer and lung adenocarcinoma cell migration and proliferation [25]. Nevertheless, the expression and functions of PPP1R14B-AS1 in breast cancer are yet to be fully explored. In this study, PPP1R14B-AS1 was evidently overexpressed in breast cancer cells. In addition, patients with breast cancer having high PPP1R14B-AS1 expression manifested worse overall survival than those with low PPP1R14B-AS1 expression. In terms of function, PPP1R14B-AS1 plays a protumorigenic role and regulates the malignant behavior of breast cancer cells.

Importantly, our research further elucidated the mechanisms underlying the aggravation of breast cancer malignancy by PPP1R14B-AS1. LncRNAs perform their biological functions by working together with different molecules. Furthermore, cytosolic lncRNAs can competitively sequester miRNAs through miRNA response elements, which decreases the availability of miRNAs and indirectly increases the levels of target mRNAs, thus functioning as a ceRNA [38,39]. Accordingly, we conducted subcellular localization analysis, which revealed that PPP1R14B-AS1 was abundant in both the nucleus and cytoplasm, with the cytoplasm carrying more PPP1R14B-AS1. This observation supports the fact that PPP1R14B-AS1 acts as a ceRNA in breast cancer cells.

The use of a bioinformatics database revealed that miR-134-3p contains complementary binding sequences for PPP1R14B-AS1. Prominently, luciferase reporter and RIP assays demonstrated the direct binding between PPP1R14B-AS1 and miR-134-3p in breast cancer cells. Moreover, results provided sufficient evidence to identify LASP1 as a direct target of miR-134-3p. Furthermore, PPP1R14B-AS1 positively regulated LASP1 in breast cancer cells by imitating miR-134-3p. Similarly, PPP1R14B-AS1, miR-134-3p, and LASP1 existed in the same RNA-induced silencing complex. In brief, PPP1R14B-AS1 can imitate miR-134-3p and antagonize the inhibitory effect of miR-134-3p on LASP1 in breast cancer cells, consequently constructing a novel PPP1R14B-AS1/miR-134-3p/LASP1 ceRNA pathway.

Aberrant expression of miR-134-3p was reported in several human cancer types [40,41]. Our current study also provided evidence that miR-134-3p was downregulated in breast cancer cells and played antitumorigenic roles, which was consistent with the results of a previous study [28]. Interestingly, mechanical experiments revealed that targeting LASP1 was essential for producing miR-134-3p-dependent cancer-inhibiting actions on breast cancer cells. LASP1, a LIM protein subfamily, not only regulates cell apoptosis and cell cycle conditions but is also implicated in the control of proliferation, migration, invasion, and epithelial–mesenchymal transition in breast cancer [42–45]. However, upstream mechanisms that cause the dysregulation of LASP1 in breast cancer are poorly defined. Our study results affirmed that LASP1 could be overexpressed by PPP1R14B-AS1 in breast cancer cells via miR-134-3p sequestration. As an inspiring observation, the knockdown of miR-134-3p or increase in LASP1 levels restored the aggressive malignant characteristic of breast cancer cells that was weakened by PPP1R14B-AS1 depletion. Thus, the miR-134-3p/LASP1 axis was found to be the effector of PPP1R14B-AS1 in breast cancer cells.

The considerable functional repertoire of lncRNAs during cancer onset and progression supports the opportunities for their therapeutic targeting [46]. At present, lncRNAs can be targeted through various methods, including transcriptional/posttranscriptional suppression, synthetic lncRNA use, and lncRNA genomic loci regulation [47]. The most prospective modality for lncRNA suppression-targeting therapy at the RNA level is based on antisense oligonucleotides [48]. After the remaining issue involving medication safety is resolved, we believe that lncRNA targeting can be widely used in the treatment of human cancers.

To summarize, our study highlights the importance of the interplay among PPP1R14B-AS1, miR-134-3p, and LASP1 in promoting the oncogenicity of breast cancer. Mechanistically, PPP1R14B-AS1 operates as a miR-134-3p sponge and relieves regulatory effect of miR-134-3p on LASP1 in breast cancer cells. Our observations may lead to the development of novel precision therapy techniques in the breast cancer treatment field.

## Data Availability

Data available with the communication author and can be provided upon reasonable request.
